# The Effect of Covert and Overt Infections on Disease Dynamics in Honey-Bee Colonies

**DOI:** 10.1007/s11538-021-00892-6

**Published:** 2021-05-06

**Authors:** Nicholas F. Britton, K. A. Jane White

**Affiliations:** Department of Mathematical Sciences and Centre for Mathematical Biology, University of Bath, Bath, UK

**Keywords:** *Apis mellifera*, Deformed-wing virus, Varroa mites, Covert and overt infections, Spontaneous transition, Directed graph

## Abstract

Viral diseases of honey bees are important economically and ecologically and have been widely modelled. The models reflect the fact that, in contrast to the typical case for vertebrates, invertebrates cannot acquire immunity to a viral disease, so they are of SIS or (more often) SI type. Very often, these diseases may be transmitted vertically as well as horizontally, by vectors as well as directly, and through the environment, although models do not generally reflect all these transmission mechanisms. Here, we shall consider an important additional complication the consequences of which have yet to be fully explored in a model, namely that both infected honey bees and their vectors may best be described using more than one infection class. For honey bees, we consider three infection classes. Covert infections occur when bees have the virus under control, such that they do not display symptoms of the disease, and are minimally or not at all affected by it. Acutely overtly infected bees often exhibit severe symptoms and have a greatly curtailed lifespan. Chronically overtly infected bees typically have milder symptoms and a moderately shortened lifespan. For the vector, we consider just two infection classes which are covert infected and overt infected as has been observed in deformed-wing virus (DWV) vectored by varroa mites. Using this structure, we explore the impact of spontaneous transition of both mites and bees from a covertly to an overtly infected state, which is also a novel element in modelling viral diseases of honey bees made possible by including the different infected classes. The dynamics of these diseases are unsurprisingly rather different from the dynamics of a standard SI or SIS disease. In this paper, we highlight how our compartmental structure for infection in honey bees and their vectors impact the disease dynamics observed, concentrating in particular on DWV vectored by varroa mites. If there is no spontaneous transition, then a basic reproduction number $R_0$ exists. We derive a condition for $R_0>1$ that reflects the complexities of the system, with components for vertical and for direct and vector-mediated horizontal transmission, using the directed graph of the next-generation matrix of the system. Such a condition has never previously been derived for a honey-bee–mite–virus system. When spontaneous transitions do occur, then $R_0$ no longer exists, but we introduce a modification of the analysis that allows us to determine whether (i) the disease remains largely covert or (ii) a substantial outbreak of overt disease occurs.

## Introduction

Colonies of the western honey bee *Apis mellifera* are almost always infected by viruses, with infection transmitted via various routes, including horizontally, vertically (from queen to egg), venereally, by physical or biological vectors, and through the environment (particularly stored food resources). One of the most common is deformed-wing virus (DWV), found in more than 80% of colonies in the latest USDA-APHIS survey in the USA. Until recently DWV was considered a minor problem (Rosenkranz et al. 2010), with infections usually without obvious pathology, called *covert* (Evans and Schwarz 2011). This is no longer the case, at least in temperate climates. Overt infection, either acute or chronic, is now common (Martin et al. 2013). Acute overt infection (with deformed wings and early death) may be seen in honey bees infected as pupae, while those infected as adults may exhibit chronic overt infection (with some cognitive deficit and possible reduced longevity). Three infectious classes of honey bees should therefore be distinguished, (i) covert, (ii) acutely overt, and (iii) chronically overt. Note that alternative terms for these different classes of infection are in wide use in the literature, but we follow the usage recommended by De Miranda and Genersch (2010) in their definitive review of DWV. A table in that paper (adapted from Hails et al. (2008)) contains a helpful summary of DWV transmission routes and outcomes.

Wilfert et al. (2016) describe the recent spread of DWV as a global epidemic. Schroeder and Martin (2012) and Martin et al. (2013) state that DWV is ‘the most likely candidate responsible for the majority of the colony losses that have occurred across the world over the last 50 years’, and ‘the key pathogen involved in colony collapse’, a conclusion backed up by other studies (Highfield et al. 2009; Genersch et al. 2010). The transformation of the disease from predominantly covert to substantially overt has been crucial. This transformation may have been exacerbated by the use of neonicotinoid pesticides (Di Prisco et al. 2013), but a likely more fundamental cause is the parasitic varroa mite *Varroa destructor* (Highfield et al. 2009; Genersch et al. 2010). These mites were originally found only in colonies of the eastern honey bee *Apis cerana*, but invaded western honey bee *Apis mellifera* populations from the middle of the 20th century onwards, probably as a consequence of commercial transportation of western honey bees to the natural range of the eastern honey bee. Colonies of western honey bees worldwide (except in Australia) are now typically infested. Compared to *Apis cerana*, which employs hygienic methods to defend itself effectively against infestations, *Apis mellifera* are badly affected, and significant infestations often lead to the death of the colony (Dietemann et al. 2012). The mites have two life stages, *phoretic* and *reproductive*. At the phoretic stage, they attach themselves to adult bees and feed on their haemolymph, occasionally moving from one host bee to the next. At the reproductive stage, they move to the brood cells of the colony where they reproduce and feed on larval bees.

Modelling the effect of pathogens on the population dynamics of invertebrates has a long history (Anderson and May 1981) and includes previous work specifically in the context of honey bees, mites and/or virus. Sumpter and Martin (2004) created a model to consider DWV assuming a fixed mite population size. Eberl’s group has concentrated on modelling acute bee paralysis virus (ABPV) (Eberl et al. 2010; Ratti et al. 2012, 2015, 2017). Others have not been specific about the virus concerned (Kang et al. 2016; Bernardi and Venturino 2016; Dénes and Ibrahim 2019), although DWV and ABPV are usually considered as examples. Dénes and Ibrahim (2019) take a different approach from others in modelling honey bees according to whether they are infested by mites, and if so whether those mites are infected by virus or not.

We shall consider the effect of the varroa mite and DWV together on the population dynamics of the western honey bee. To do this, we require some insight into the interaction between mites and DWV. There is strong evidence that the virus replicates within the mite (Kevan et al. 2006; Gisder et al. 2009). So the virus may be ingested by a mite at the phoretic stage (in the haemolymph of a covertly or overtly infected adult bee), may replicate within the mite, and may be passed on at high levels to a larval bee in a brood cell when the mite is at the reproductive stage (Yue and Genersch 2005). Typically, the larval bee then shows acute overt symptoms of DWV at the adult stage, with characteristically deformed wings, and dies within 2 or 3 days of emergence (Gisder et al. 2009). The mite acts not simply as a physical vector but as a biological vector for the virus (Kevan et al. 2006), and amplifies the effects of the pathogen from covert to overt. The mites themselves may be infected with DWV at a low or at a high level, depending on whether replication has occurred or not. This determines the effect that they have on their honey-bee hosts, and it is necessary in a model to distinguish these two infectious classes. We shall call infections at a low-level covert, and at a high-level overt, although the mite does not seem to suffer symptoms even from high-level infections.

The most important quantity for an infectious disease is $R_0$, the basic reproduction number, which determines whether and how widely disease will spread if introduced into an initially disease-free population. For the first time, we shall derive expressions to determine whether $R_0>1$ in a dynamically varying honey-bee–mite–virus system, when a disease-free steady state exists. When there is no such steady state we shall introduce a new analysis that determines whether a disease remains predominantly covert (as DWV did before varroa mites became established) or breaks out and becomes a substantially overt disease, leading in the case of DWV to widespread colony losses. To do this, we build our mathematical model sequentially. In the following section, we propose a model to describe the interaction between honey bees and mites. Having established the key dynamic properties of that system, we extend our model to incorporate viral infection which allows us to derive expressions for $R_0$ as described above. In the conclusions, we discuss the significance of the work presented, both the approach to calculating $R_0$ and the results in the context of understanding the importance of overt and covert infections and associated spontaneous transitions from covert to overt infections within mite and honey-bee populations.

## Modelling Interactions Between Honey Bees and Mites

In the absence of viral infection, we define *N*(*t*) and *M*(*t*) to be the number of honey bees in a colony and mites in that colony at time *t*. Following previous published work, we make the following model choices and assumptions:

- Bee production depends on the number of workers in the colony since they are necessary to care for the brood and to gather resources for the colony. Consequently, we assume that production *h*(*N*) is a saturating function of colony size following (Eberl et al. 2010; Khoury et al. 2011, 2013; Kang et al. 2016) and choose the functional form to follow Eberl’s group (Ratti et al. 2012, 2015, 2017):
  $$\begin{aligned} h(N) = \displaystyle \frac{N^2}{A^2 +N^2} \end{aligned}$$
  where $A^2$ is a positive constant.
- We assume that the death rate of bees in a colony due to parasitism by mites is directly proportional to the number of mites in the environment. This differs from previous authors who have used a mass action assumption (Ratti et al. 2012, 2015, 2017; Kang et al. 2016). It was chosen such that the per capita honey-bee death rate due to parasitism is proportional to the number of mites per honey bee which we interpret as a measure of stress on the bee that leads to its increased death rate.
- Mites physically attach themselves to their hosts and so we follow Eberl’s group and use a Leslie–Gower approach (Pielou 1977) by assuming that mites grow logistically during the summer months with a carrying capacity that is proportional to the size of the host colony (Ratti et al. 2012, 2015, 2017). In the winter, we assume that mites die at a constant per capita rate.

Using these assumptions, our model for the honey-bee–mite interactions is:

1 $$\begin{aligned} \begin{aligned} \frac{\mathrm{d}N}{\mathrm{d}t} =&f(N,M) = \alpha h(N) - \mu N - \gamma M,\\ \frac{\mathrm{d}M}{\mathrm{d}t} =&g(N,M) = {\left\{ \begin{array}{ll} r M(1 - M/(kN)) &{} \text {if } r>0,\\ - s M &{} \text {if } r=0, \end{array}\right. } \end{aligned} \end{aligned}$$

with $\alpha >0$ and $r>0$ in the growing season, $\alpha =r=0$ in the winter. The remaining model parameters $\mu$, $\gamma$, *k* and *s* are all positive constants which take the following meaning: $\mu$ is the per capita natural death rate of honey bees; $\gamma M$ is the parasite-related death rate of the bees; *r* is the intrinsic growth rate of the mites which grow logistically with a carrying capacity *kN*; the parameter *s* denotes the per capita death rate of mites in the winter period. The parameters vary with time in a temperate climate, even within the growing season, however for the work presented here, we assume that the parameters are constant and focus on the summer period.

### Honey-Bee Dynamics in the Absence of Mites

If $M(0)=0$, then $M(t)=0$ for all *t*, so the *N* equation becomes

2 $$\begin{aligned} \begin{aligned} \frac{\text {d}N}{\text {d}t} =&f_0(N) = f(N,0) = \alpha h(N) - \mu N\\ =&- N \frac{\mu N^2 - \alpha N + \mu A^2}{N^2 + A^2}. \end{aligned} \end{aligned}$$

**Fig. 1:**
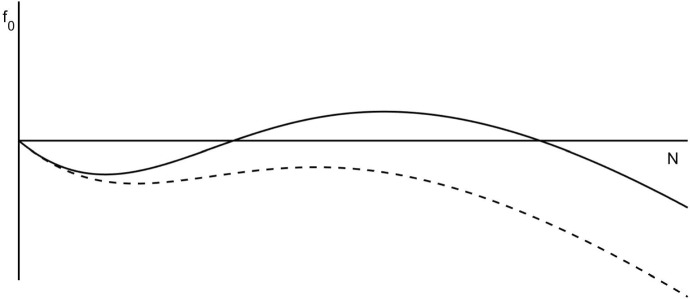
The function $f_0$, describing the growth rate of honey bees within a colony in the absence of mites as given in (2). The solid line corresponds to the case $\alpha >2\mu A$ while the dashed line is for $\alpha <2\mu A$

The function $f_0$ is as shown in Fig. 1, for $\alpha <2\mu A$ and $\alpha >2\mu A$.

**Fig. 2:**
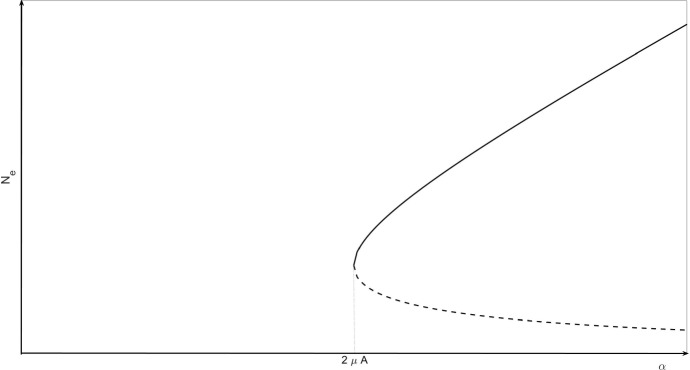
Bifurcation diagram showing how the equilibrium values, $N_e$, of (2) and their stability vary as the parameter $\alpha$ (corresponding to the growth in colony size) increases. For small $\alpha$ the colony cannot be maintained; once the critical threshold $\alpha = 2 \mu A$ is exceeded, the colony exhibits bistable dynamics. The solid line corresponds to locally stable equilibria, $N_e=0$ and $N_e = {\hat{N}}_2$ while the dotted line represents an unstable equilibrium, $N_e={\hat{N}}_1$

The bifurcation structure for this system is easy to analyse. It has a stable steady state at $N=0$. Two other steady states, $0<{\hat{N}}_1<{\hat{N}}_2$, the first unstable and the second stable, appear by a saddle–node bifurcation for $\alpha >2\mu A$. We may write ${\hat{N}}_i=An_i(\beta )$ for $i=1,2$, where $\beta =\alpha /(\mu A)$, and

3 $$\begin{aligned} n_1(\beta ) = \frac{1}{2}(\beta - \sqrt{\beta ^2 - 4}), \qquad n_2(\beta ) = \frac{1}{2}(\beta + \sqrt{\beta ^2 - 4}), \end{aligned}$$

real and positive for $\beta >2$, or $\alpha >2\mu A$. The bifurcation diagram is as shown in Fig. 2.

### Analysis of the Full System

During the growing season, our model system (1) can be written as,

4 $$\begin{aligned} \begin{aligned} \frac{\mathrm{d}N}{\mathrm{d}t} =&f(N,M) = \alpha h(N) - \mu N - \gamma M,\\ \frac{\mathrm{d}M}{\mathrm{d}t} =&g(N,M) = r M\left( 1 - \frac{M}{kN}\right) . \end{aligned} \end{aligned}$$

The system has a singularity at $N=0$. But the transformation to *N*, $\varphi =M/N$ leads to

$$\begin{aligned} \begin{aligned} \frac{\mathrm{d}N}{\mathrm{d}t} =&\alpha h(N) - \mu N - \gamma N \varphi ,\\ \frac{\mathrm{d}\varphi }{\mathrm{d}t} =&r\varphi (1 - \varphi /k) - \alpha \varphi h(N)/N + \mu \varphi - \gamma \varphi ^2, \end{aligned} \end{aligned}$$

which has no singularities and is in Kolmogorov form, so that the positive quadrant of $(N,\varphi )$ space is positively invariant. It follows that the positive quadrant of (*N*, *M*) space is positively invariant, despite the singularity in *g* and the $-\gamma M$ term in the *N* equation.

The nullclines and steady states for this system are as follows. For $g(N,M)=0$, either $M={\hat{G}}(N)=0$ or $M=G^*(N)=kN$. For $f(N,M)=0$, $M=F(N)=(1/\gamma )f_0(N)$, where $f_0$ is as in (2). The nullclines $M=F(N)$ and $M={\hat{G}}(N)=0$ intersect where

$$\begin{aligned} {\hat{Q}}(N) = \mu N^2 - \alpha N + \mu A^2 = 0. \end{aligned}$$

The roots of the quadratic ${\hat{Q}}(N)=0$ are given as before by ${\hat{N}}_i=An_i(\beta )$, where $\beta =\alpha /(\mu A)$ and the functions $n_i$ are defined in (3), real and positive if $\beta >2$, $\alpha >2\mu A$. The nullclines $M=F(N)$ and $M=G^*(N)=kN$ intersect where

$$\begin{aligned} Q^*(N) = (\mu + k\gamma )N^2 - \alpha N + (\mu + k\gamma )A^2 = 0. \end{aligned}$$

This quadratic is the same as ${\hat{Q}}$ above but with $\mu$ replaced by $\mu +k\gamma$. Its roots are given by $N_i^*=An_i(\beta )$, where the functions $n_i$ are again as in (3) but now $\beta =\alpha /((\mu +k\gamma )A)$, and are real and positive if $\beta >2$, $\alpha >2(\mu +k\gamma )A$.

There are therefore three cases.

(i) $\alpha <2\mu A$: the curve $M=F(N)$ never enters the positive quadrant.
(ii) $2\mu A<\alpha <2(\mu +k\gamma )A$: the curve $M=F(N)$ is in the positive quadrant between ${\hat{N}}_1$ and ${\hat{N}}_2$, but never intersects the straight line $M=G^*(N)=kN$.
(iii) $\alpha >2(\mu +k\gamma )A$: the curve $M=F(N)$ is in the positive quadrant between ${\hat{N}}_1$ and ${\hat{N}}_2$, and intersects the straight line $M=G^*(N)$ at $N_1^*$ and $N_2^*$. It is clear that ${\hat{N}}_1<N_1^*<N_2^*<{\hat{N}}_2$.

We shall consider each case in turn.

**Case (i)**, $$\alpha <2\mu A$$.

The set *D* given by

$$\begin{aligned} D = \left\{ (N,M) \,|\, 0<N<C, \, 0<M<kC \, \right\} \end{aligned}$$

is positively invariant for any positive constant *C*, so that the origin is globally asymptotically stable. The honey-bee production rate is not under any circumstances sufficient to outweigh the death rate. Henceforth, we shall assume that $\alpha >2\mu A$.

**Case (ii)**, $$2\mu A<\alpha <2(\mu +k\gamma )A$$

**Fig. 3:**
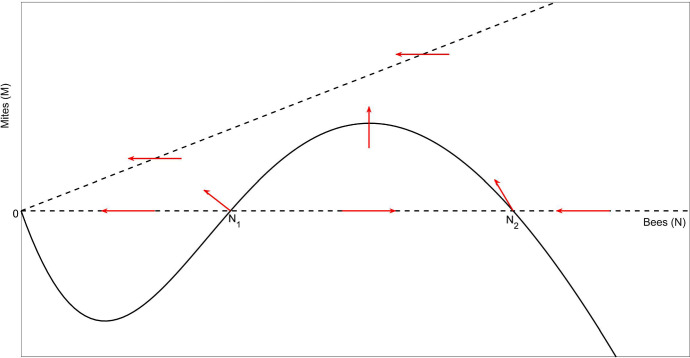
The phase plane for Case (ii) with medium honey-bee production rate, $2\mu A<\alpha <2(\mu +k\gamma )A$. The dashed lines represent the *M* nullclines along which $g(N,M)=0$ and the solid line represents the *N* nullcline along which $f(N,M)=0$. The red arrows show the direction of solution trajectories as time increases. The origin is globally asymptotically stable in the strictly positive quadrant

There are no periodic solutions in $\mathbb {R}_+^2$ (since there are no steady states there). No trajectories approach $({\hat{N}}_1,0)$ and the only trajectories to approach $({\hat{N}}_2,0)$ do so along the *N* axis. The set *D* given by

$$\begin{aligned} D = \left\{ (N,M) \,|\, 0<N<C, \, 0<M<kC \, \right\} \end{aligned}$$

is positively invariant for any constant $C>\hat{N_2}$ (and for $0<C<\hat{N_1}$). By the Poincaré–Bendixson theorem, the origin is globally asymptotically stable (in the strictly positive quadrant). This case is shown graphically in Fig. 3.

**Case (iii)**, $$\alpha >2(\mu +k\gamma )A$$.

There are semi-trivial (mite-free) steady states at $(\hat{N_1},0)$ and $(\hat{N_2},0)$, and non-trivial steady states at $(N_1^*,M_1^*)$ and $(N_2^*,M_2^*)$, where $M_1^*=kN_1^*$ and $M_2^*=kN_2^*$.

The character of each steady state is determined by the Jacobian matrix *J*, where

$$\begin{aligned} J(N,M) = \begin{pmatrix} \alpha h'(N) - \mu &{} - \gamma \\ (rM^2)/(kN^2) &{} r\left( 1 - (2M)/(kN)\right) \end{pmatrix}. \end{aligned}$$

For $$({\hat{N}}_1,0)$$ and $$({\hat{N}}_2,0)$$,

$$\begin{aligned} J({\hat{N}},0) = \begin{pmatrix} \alpha h'({\hat{N}}) - \mu &{} - \gamma \\ 0 &{} r \end{pmatrix}, \end{aligned}$$

with eigenvalues $\alpha h'({\hat{N}})-\mu$ and *r*. The slope of the curve $M=F(N)$ is $F'(N)=(\alpha h'(N)-\mu )/\gamma$. Hence, $\alpha h'({\hat{N}}_1)-\mu >0$, $({\hat{N}}_1,0)$ is an unstable node. And $\alpha h'({\hat{N}}_2)-\mu <0$, $({\hat{N}}_2,0)$ is a saddle point.

For $$(N_1^*,M_1^*)$$ and $$(N_2^*,M_2^*)$$,

$$\begin{aligned} J^* = J(N^*,M^*) = \begin{pmatrix} \alpha h'(N^*) - \mu &{} - \gamma \\ r k &{} - r \end{pmatrix}. \end{aligned}$$

At $(N_1^*,M_1^*)$, the slope of the curve $M=F(N)$ is greater than *k*, $\alpha h'(N_1^*)-\mu >k\gamma$, so

$$\begin{aligned} \det J_1^* = \det J(N_1^*,M_1^*) = -r(\alpha h'(N_1^*) - \mu ) + r k \gamma < 0, \end{aligned}$$

and $(N_1^*,M_1^*)$ is a saddle point.

**Fig. 4:**
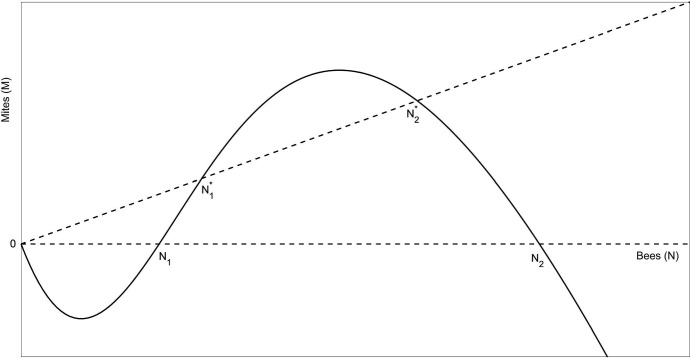
The phase plane for Case (iii) with high honey-bee production rate, $2(\mu +k\gamma )A<\alpha$. As above, the dashed lines represent the *M* nullclines along which $g(N,M)=0$ and the solid line represents the *N* nullcline along which $f(N,M)=0$. The phase plane exhibits bistable properties such that there are two locally stable equilibrium points, the origin and $N^*_2$, separated by an unstable equilibrium $N^*_1$

Similarly $\alpha h'(N_2^*)-\mu <k\gamma$, so $\det J_2^*=\det J(N_2^*,M_2^*)>0$, and $(N_2^*,M_2^*)$ is a stable or unstable node or focus. Also, ${\text {tr}}J_2^*=\alpha h'(N_2^*)-\mu -r<k\gamma -r$, so ${\text {tr}}J_2^*<0$ and $(N_2^*,M_2^*)$ is stable as a solution of the disease-free system if *r* is sufficiently large, and in particular if

5 $$\begin{aligned} r > k\gamma . \end{aligned}$$

It is not globally stable because of the Allee effect built into the model. In fact, the analysis of case (ii) restricted to the positively invariant set *D* given by

$$\begin{aligned} D = \left\{ (N,M) \,|\, 0<N<C, \, 0<M<kC \, \right\} \end{aligned}$$

with $0<C<\hat{N_1}$ shows that the origin is still asymptotically stable (but of course no longer globally asymptotically stable). This case is shown graphically in Fig. 4.

## Modelling Viral Infection within the Honey-Bee and Mite Ecosystem

Central to our assumption that infection classes in the bee populations should be compartmentalised we divide the bee colony according to infection status using the empirical evidence that very few bees in a virus-infected colony are uninfected (Yue and Genersch 2005). At time *t*, individual bees may be in one of three states: covertly infected *X*(*t*), chronically overtly infected *Y*(*t*) or acutely infected *Z*(*t*) such that

$$\begin{aligned} X+Y+Z = N. \end{aligned}$$

Similarly, very few mites in a DWV-infected colony are virus-free (Anguiano-Baez et al. 2016), and we neglect them. The virus titre in infected mites varies from around $10^8$ particles to $10^{10}$ or as much as $10^{12}$ viral genome equivalents (Gisder et al. 2009), depending on whether or not virus replication has taken place in the mite. Therefore, we create two compartments for the mite population at time *t*: covert infected *U*(*t*) and overt infected *V*(*t*) such that

$$\begin{aligned} U+V=M. \end{aligned}$$

Once overtly infected, bees and mites remain overtly infected throughout their life.

**Fig. 5:**
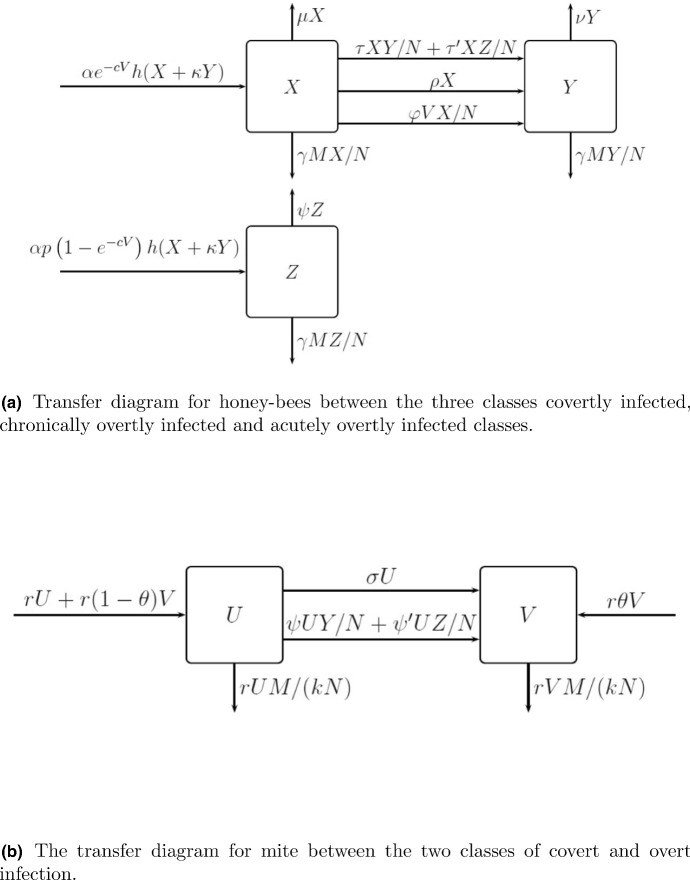
Transfer diagrams showing the flow between infection classes separately for the honey bees, shown in (**a**), and mites, shown in (**b**). Justification of the different components and their functional forms is given in the text

Figure 5a and b shows the transfer between these classes separately for the bees and the mites and should be used as an aide memoire as we now describe the demographic and infection processes that we combine together to describe viral infection within the honey-bee and mite ecosystem.

**Honey-bee production** The infection status of adult bees emerging from brood cells will clearly impact the model dynamics. Several different approaches have been taken in the literature each arising from different underlying assumptions (Sumpter and Martin 2004; Eberl et al. 2010; Ratti et al. 2012, 2017; Bowen-Walker et al. 1999; Bernardi and Venturino 2016; Kang et al. 2016). We follow an approach that is closest to Sumpter and Martin (2004) who model the infection status of adult bees emerging from brood cells phenomenologically, using data suggesting that phoretic mites entering the reproductive stage (whose infection status is part of the model) are Poisson distributed among brood cells (Martin 1995; Salvy et al. 1999). This phenomenon directly impacts the class into which newborn bees emerge: with probability $e^{-cV}$, where *c* is a positive constant, a newborn is covertly infected and contributes to population *X* and with probability $1-e^{-cV}$, they are acutely infected and contribute to population *Z*. In the case of acute infection, there is an additional chance of mortality and so the birth rate of acutely infected bees is reduced by a factor *p*, $0 \le p < 1$, compared with the covertly infected newborns.

We also assume that infection status of the queen and workers impacts their ability to produce viable eggs in the following ways:

- The queen bee is covertly infected, and transmits the covert but not the overt disease to her offspring;
- Acutely overtly infected bees do not contribute to production;
- Chronically overtly infected bees make a reduced contribution with parameter $\kappa$ (Hails et al. 2008; Sumpter and Martin 2004).

**Honey-bee mortality** The natural per capita death rates $\mu$, $\nu$ and $\zeta$ of *X*, *Y* and *Z* bees depend on the bee’s infection status, such that $\mu<\nu <\zeta$ (Hails et al. 2008). Mite-related honey-bee death is assumed to be independent of infection status of the bee with rate parameter $\gamma$ and assuming a frequency-dependent functional form.

**Mite production and mortality** There is no evidence in the literature that mites are affected by DWV and so we assume that there is no negative impact on mites that have the virus. Consequently, mite dynamics are assumed to follow those described in Sect. 2 with an intrinsic growth rate *r*, carrying capacity *kN* and winter mortality rate *s*, independent of infection status. Overt disease may be transmitted vertically and so we assume that a fraction $\theta$ of new infections from overtly infected mites produce overtly infected mites; the remainder are covertly infected.

**Rates of infection transmission for adult bees and mites** Transmission of infection for both adult bees and mites results in movement between covert and overt infected status both due to interactions between individuals and as a result of spontaneous transition where growth in viral load within an individual results in a change in the status. We assume the following transmission routes:

- Horizontal transmission of chronic overt infection from one adult bee to another does occur (Hails et al. 2008). Disease may be transmitted in its chronic overt form from a chronically overtly or an acutely overtly infectious bee to a covertly infectious bee, with frequency-dependent infectious contact parameter $\tau$ and $\tau ^{\prime }$, respectively. This may be a minor route of transmission.
- Disease may be transmitted from mites to bees and vice versa, with $\varphi$ the infectious contact parameter from mites to bees, $\psi$ (respectively, $\psi ^{\prime }$ for acutely overtly infected bees) that from bees to mites, with frequency-dependent transmission.
- Spontaneous transition from covert to chronic overt disease may occur in an adult bee (Sumpter and Martin 2004; Nazzi et al. 2012; Di Prisco et al. 2013), by viral replication. We assume this happens at a per capita rate $\rho$. Such transitions may be rare. We assume that spontaneous transitions from covert to acute overt disease in larval bees do not occur, as acutely overtly infected bees are not observed in mite-free colonies.
- Spontaneous transition from covert to overt infection has been observed in mites (Kevan et al. 2006; Yue and Genersch 2005; Gisder et al. 2009). We include them in our model assuming a per capita rate of transition $\sigma$. Autonomous spontaneous transitions in the opposite direction may possibly occur but the evidence for them is less clear and we have not included them. We note that spontaneous transition in bees in particular may be rare.

Combining these model components and assumptions, we present our model system (for the spring, summer and autumn periods) takes the form:

6 $$\begin{aligned} \begin{aligned} \frac{\mathrm{d}X}{\mathrm{d}t} =&\alpha e^{-cV} h(X + \kappa Y) - \mu X - \gamma M \frac{X}{N} - \rho X - \tau X \frac{Y}{N} - \tau ' X \frac{Z}{N}- \varphi \frac{X}{N} V, \\ \frac{\mathrm{d}Y}{\mathrm{d}t} =&\rho X + \tau X \frac{Y}{N} + \tau ' X \frac{Z}{N} - \nu Y - \gamma M \frac{Y}{N} + \varphi \frac{X}{N} V, \\ \frac{\mathrm{d}Z}{\mathrm{d}t} =&\alpha p(1 - e^{-cV}) h(X + \kappa Y) - \zeta Z - \gamma M \frac{Z}{N}, \\ \frac{\mathrm{d}U}{\mathrm{d}t} =&r U + r(1 - \theta )V - r U \frac{M}{kN} - \sigma U - \psi U \frac{Y}{N} - \psi ' U \frac{Z}{N}, \\ \frac{\mathrm{d}V}{\mathrm{d}t} =&r \theta V - r V \frac{M}{kN} + \sigma U + \psi U \frac{Y}{N} + \psi ' U \frac{Z}{N}, \end{aligned} \end{aligned}$$

where $$h(N)=N^2/(A^2+N^2)$$ and $$\mu<\nu <\zeta $$.

In winter, there is no brood, so $\alpha =0$, $r=0$, and the equations for *U* and *V* are replaced by

$$\begin{aligned} \frac{\mathrm{d}U}{\mathrm{d}t} = - s U, \;\;\; \frac{\mathrm{d}V}{\mathrm{d}t} = - s V. \end{aligned}$$

### Analysis of the Mite-Free Model for Bees and Disease

Acutely overtly infected (Z) bees are only produced by vector-borne transmission, so we take $Z=0$. The X and Y equations are given by

7 $$\begin{aligned} \begin{aligned} \frac{\mathrm{d}X}{\mathrm{d}t} =&\alpha h(X + \kappa Y) - \mu X - \rho X - \tau X \frac{Y}{N}, \\ \frac{\mathrm{d}Y}{\mathrm{d}t} =&- \nu Y + \rho X + \tau X \frac{Y}{N}. \end{aligned} \end{aligned}$$

We seek steady states $(X^*,Y^*)$, with $X^*+Y^*=N^*$, and $x^*=X^*/N^*$, $y^*=Y^*/N^*$, and $x^*+y^*=1$. Then, from the Y equation,

8 $$\begin{aligned} 0 = - \nu y^* + \rho (1 - y^*) + \tau y^*(1 - y^*), \end{aligned}$$

a quadratic equation with one negative root and one root between 0 and 1. From now on, let $y^*$ denote the root between 0 and 1 and let $x^*=1-y^*$, also between 0 and 1. The sum of the equations in (7) at steady state then gives

9 $$\begin{aligned} \alpha h(\xi N^*) = \mu \eta N^*, \end{aligned}$$

where $\xi =x^*+\kappa y^*<1$, $\mu \eta =\mu x^*+\nu y^*>\mu$, or $\eta >1$, since $\kappa <1$, $\nu >\mu$. (Note that the expressions $\xi$ and $\eta$ may be given explicitly in terms of the parameters of the system.) This is just a rescaled version of the standard equation $\alpha h(N)=\mu N$, from (2), with solutions given by (3). We can therefore immediately give the solutions as

10 $$\begin{aligned} N_i^* = \frac{A}{\xi } n_i \left( \frac{\alpha \xi }{\mu \eta A} \right) , \end{aligned}$$

positive and realistic for $\alpha \xi >2\mu \eta A$. The expression simplifies if Y bees are not dysfunctional compared to X bees, $\kappa =1$ and $\nu =\mu$, since then $\xi =1$ and $\eta =1$. Unsurprisingly, the more dysfunctional Y bees are compared to X bees, in other words the smaller $\kappa$ is and/or the larger $\nu$ is, the larger the production rate $\alpha$ has to be to prevent the colony collapsing to zero. We shall now consider three special cases.

#### Case (i): No Horizontal Transmission from Bee to Bee, $\tau = 0$

The assumption that $\tau =0$ is an assumption that all previous models except Kang et al. (2016) have made. The resulting Y equation is given by

$$\begin{aligned} \frac{\mathrm{d}Y}{\mathrm{d}t} = \rho X - \nu Y, \end{aligned}$$

so that $X^*=\nu N^*/(\nu +\rho )$, $Y^*=\rho N^*/(\nu +\rho )$. There is no Y-free steady state, except (0, 0). Every colony with $\rho >0$ contains bees with overt disease, although if $\rho$ is small (see cases (ii) and (iii) below) there are very few of them, and if $\rho =0$ there are none.

#### Case (ii): No Spontaneous Transition in Bees, $\rho =0$

All previous models have made this assumption, usually with $\tau =0$ as well. It is likely to be at least a good approximation, unless the bees’ immune system has been compromised by mites or neonicotinoids (Di Prisco et al. 2013). The transition from X to Y occurs by contact instead of spontaneously, and the equations are

11 $$\begin{aligned} \begin{aligned} \frac{\mathrm{d}X}{\mathrm{d}t} =&\alpha h(X + \kappa Y) - \mu X - \tau X \frac{Y}{N} = f(X,Y),\\ \frac{\mathrm{d}Y}{\mathrm{d}t} =&- \nu Y + \tau X \frac{Y}{N} = g(X,Y). \end{aligned} \end{aligned}$$

Seeking steady states $(X^*,Y^*)=(N^*x^*,N^*y^*)$, as before, Eq. (8) becomes $-\nu y^*+\tau y^*(1-y^*)=0$, so (a) $y^*=0$ or (b) $x^*=1-y^*=\nu /\tau$, $y^*=1-\nu /\tau$, realistic if and only if $\nu <\tau$. For alternative (b), the quantity $N^*$ may then be calculated from the sum of the X and the Y equation in the usual way, leading to Eq. (9) with $\xi =\kappa +(1-\kappa )\nu /\tau$, $\mu \eta =\mu \nu /\tau +\nu (1-\nu /\tau )$, and two solutions given by Eq. (10), positive and realistic for $\alpha \xi >2\mu \eta A$. Let us now consider alternative (a), with $Y^*=y^*=0$. The steady-state X equation then becomes very familiar, $\alpha h(X^*)=\mu X^*$, with two solutions $X^*=N^*={\hat{N}}_i=An_i(\alpha /(\mu A))$ for $i=1,2$, realistic for $\alpha >2\mu A$.

One overt-disease-free steady state always exists, the trivial steady state (0, 0), and there are two more given by $({\hat{N}}_1,0)$ and $({\hat{N}}_2,0)$ as long as $\alpha >2\mu A$. There are also two steady states with overt disease, $(X_1^*,Y_1^*)=(N_1^*x^*,N_1^*y^*)$ and $(X_2^*,Y_2^*)=(N_2^*x^*,N_2^*y^*)$, described above, as long as both $\nu <\tau$ and $\alpha \xi >2\mu \eta A$.

The overt-disease-free steady states appear by a saddle–node bifurcation at $\alpha =2\mu A$, and we know from bifurcation theory (as in Sect. 2.1) that $({\hat{N}}_1,0)$ is unstable and $({\hat{N}}_2,0)$ stable as solutions of the Y-free system. We wish to test whether $({\hat{N}}_2,0)$ is stable as a solution of the full system (11). So consider introducing an overtly infected Y bee (a *primary*) into the system at the steady state $({\hat{N}}_2,0)$. While the primary is in the Y compartment it makes infectious contacts at rate $\tau X/N = \tau$ at the steady state. Bees in the Y compartment leave it at per capita rate $\nu$, so they spend time $1/\nu$ in the compartment on average. Hence, the primary makes an expected $R_0^-=\tau /\nu$ infectious contacts. $R_0^-$ is called the *basic reproduction number*. There is a threshold $R_0^-=1$ for spread of overt disease: it spreads if $R_0^->1$ but not if $R_0^-<1$. So the disease spreads if $\tau >\nu$, but not if $\tau <\nu$. If $\tau <\nu$ there is no steady state with overt disease. If $\tau >\nu$ and $\alpha \xi <2\mu \eta A$, there is still no steady state with overt disease. The trajectory starting with a perturbation from $({\hat{N}}_2,0)$ must tend to (0, 0). If $\tau >\nu$ and $\alpha \xi >2\mu \eta A$ there are two steady states with overt disease. The trajectory starting with a perturbation from $({\hat{N}}_2,0)$ tends either to (0, 0) or to $(X_2^*,Y_2^*)$, unless there is a Hopf bifurcation allowing periodic solutions about $(X_2^*,Y_2^*)$. This system is essentially an SI model, and its bifurcation behaviour is quite different from the system with spontaneous transition, $\rho >0$. In particular, there is a threshold value $R_0^-=\tau /\nu =1$ below which overt disease cannot exist. The notation $R_0^-$ emphasises that this is the basic reproduction number *in the absence of mites*. The difference between this and the corresponding basic reproduction number $R_0$ with mites present that we shall discuss in the next section is the basis for the different behaviours of infected bee colonies with and without mites, and for an explanation of the grievous effect of mites on bee colonies.

#### Case (iii): Very Little Spontaneous Transition in Bees, $\rho$ Small

This is likely to be a good assumption unless the bees’ immune systems are compromised, which could be because of high levels of infestation by mites (Nazzi et al. 2012) or because of high levels of neonicotinoid pesticides in the environment (Di Prisco et al. 2013). Equation (8) still holds, with solutions $y^*=\rho /(\nu -\tau )+O(\rho ^2)$, $y^*=1-\nu /\tau +O(\rho )$. If $\nu >\tau$ the first of these is realistic, and the corresponding steady states have low ($O(\rho )$) prevalence of overt disease, while if $\nu <\tau$ the second is, and the steady states have *O*(1) prevalence. Strictly speaking there is no basic reproduction number, but $R_0=\tau /\nu$ still has a role to play: overt disease is maintained at a low level for $R_0^-<1$ but not for $R_0^->1$.

### The Complete System

We shall now analyse the complete system (6), with honey bees, varroa mites, and DWV. We recall that in spring, summer and autumn, the model equations are given by

$$\begin{aligned} \begin{aligned} \frac{\mathrm{d}X}{\mathrm{d}t} =&\alpha e^{-cV} h(X + \kappa Y) - \mu X - \gamma M \frac{X}{N} - \rho X - \tau X \frac{Y}{N} - \tau ' X \frac{Z}{N} - \varphi \frac{X}{N} V,\\ \frac{\mathrm{d}Y}{\mathrm{d}t} =&\rho X + \tau X \frac{Y}{N} + \tau ' X \frac{Z}{N} - \nu Y - \gamma M \frac{Y}{N} + \varphi \frac{X}{N} V,\\ \frac{\mathrm{d}Z}{\mathrm{d}t} =&\alpha p(1 - e^{-cV}) h(X + \kappa Y) - \zeta Z -\gamma M \frac{Z}{N},\\ \frac{\mathrm{d}U}{\mathrm{d}t} =&r U + r(1 - \theta )V - r U \frac{M}{kN} - \sigma U - \psi U \frac{Y}{N} - \psi ' U \frac{Z}{N},\\ \frac{\mathrm{d}V}{\mathrm{d}t} =&r \theta V - r V \frac{M}{kN} + \sigma U + \psi U \frac{Y}{N} + \psi ' U \frac{Z}{N}. \end{aligned} \end{aligned}$$

#### Case (i): No Spontaneous Transition in Bees and Mites, $\rho = \sigma = 0$

Motivated by our analysis of the mite-free system, we shall start by assuming that there is no spontaneous transition to overt disease, $\rho =\sigma =0$. Let $\alpha >2A(\mu +k\gamma )$ (so $N_2^*$ exists) and let *r* be so large that $(N_2^*,M_2^*)$ is stable as a solution of the disease-free system (e.g. $r>k\gamma$, see Eq. (5)).

We shall analyse this system using the next-generation matrix method (Diekmann et al. 1990; Van den Driessche and Watmough 2008). The next-generation matrix *K* is a generalisation of the basic reproduction number $R_0$. In this case, it is 3$\times$3, with rows and columns related to the three overt disease classes Y, Z, and V, which are zero in an overt-disease-free steady state. It is given by $K=FD^{-1}$, where *F* is the matrix whose component $F_{ij}$ gives the rate at which individuals in overtly infected class *i* arise through infection by those in class *j* near the steady state, and $D_{ii}$ denotes the rate at which those in class *i* leaves that class through death. (In general, we would have to consider those that entered a particular infected class by transition from another infected class, and those that left an infected class other than through death, but there are no such processes in this system.) Then, $K_{ij}$ denotes the number of disease offspring a primary of class *j* produces in class *i* throughout the life-time of its disease. It may be shown that the basic reproduction number $R_0$ for the system at the steady state is given by the largest eigenvalue of *K*. Here, the next-generation matrix *K* about the overt-disease-free steady state $S_2^*$ is given by

12 $$\begin{aligned} K =&\begin{pmatrix} K_{YY} &{} K_{YZ} &{} K_{YV} \\ K_{ZY} &{} K_{ZZ} &{} K_{ZV} \\ K_{VY} &{} K_{VZ} &{} K_{VV} \end{pmatrix}\nonumber \\ =&\begin{pmatrix} \tau &{} \tau ' &{} \varphi \\ 0 &{} 0 &{} \alpha p c h_2^* \\ k\psi &{} 0 &{} r\theta \end{pmatrix} \begin{pmatrix} 1/(\nu +k\gamma ) &{} 0 &{} 0 \\ 0 &{} 1/(\zeta +k\gamma ) &{} 0 \\ 0 &{} 0 &{} 1/r \end{pmatrix}\nonumber \\ =&\begin{pmatrix} \tau /(\nu +k\gamma ) &{} \tau '/(\zeta +k\gamma ) &{} \varphi /r \\ 0 &{} 0 &{} \alpha p c h_2^*/r \\ k\psi /(\nu +k\gamma ) &{} k\psi '/(\zeta +k\gamma ) &{} \theta \end{pmatrix}, \end{aligned}$$

where we have written $h_2^*=h(N_2^*)$. The characteristic polynomial *P* of *K* is given by

13 $$\begin{aligned} P(\lambda ) =&-\lambda (\lambda - \theta )\left( \lambda - \frac{\tau }{\nu + k\gamma }\right) + \left( \frac{k\varphi \psi }{r(\nu + k\gamma )} + \frac{\alpha pch_2^*k\psi '}{r(\zeta + k\gamma )} \right) \lambda \nonumber \\&+ \frac{\alpha pch_2^*}{r} \left( \frac{k\psi \tau '-k\psi '\tau }{(\nu + k\gamma )(\zeta + k\gamma )} \right) . \end{aligned}$$

The roots of the characteristic equation $P(\lambda )=0$ are the eigenvalues of *K*. This is a cubic with a negative $\lambda ^3$ coefficient, so it has at least one root greater than 1 (and hence $R_0>1$) if $P(1)>0$. Hence, $P(1)>0$ is sufficient for $R_0>1$, but it is not necessary: a cubic *P* with $P(1)<0$ may have two roots greater than 1. In that case, it may be easier to check whether $R_0>1$ by considering circuits of transmission of overt infection in a directed graph.

**Fig. 6:**
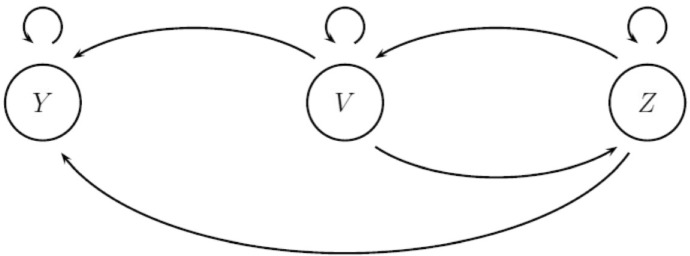
Arcs of transmission for overt infection. There is an arc (or directed edge) from *i* to *j* if the component $K_{ij}$ in the next-generation matrix *K* is positive. The arc weights (not shown in the diagram) are the components $K_{ij}$

Arcs of transmission are shown in Fig. 6. We shall define a (single-generation) circuit of transmission as a sequence of arcs starting from node *i* and ending at node *i* but not otherwise visiting node *i*. The simplest circuits of transmission are arcs direct from a node to itself: mites V infecting other mites (which then enter V) vertically, V$\rightarrow$V; and bees Y infecting other bees (which then enter Y) horizontally, Y$\rightarrow$Y. We shall denote the partial basic reproduction numbers for these processes as

$$\begin{aligned} R_0^V=K_{VV}=\theta \end{aligned}$$

and

$$\begin{aligned} R_0^Y=K_{YY}=\tau /(\nu +k\gamma ). \end{aligned}$$

The circuits of length 2 are Y bees infecting mites (which then enter V) infecting other bees (which then enter Y), Y$\rightarrow$V$\rightarrow$Y, and Z bees infecting mites (which then enter V) infecting other bees (which then enter Z), Z$\rightarrow$V$\rightarrow$Z. We shall denote the partial basic reproduction numbers for these processes as $R_0^{YV}$ and $R_0^{VZ}$, where

$$\begin{aligned} (R_0^{YV})^2=K_{YV}K_{VY}=(k\varphi \psi )/(r(\nu +k\gamma )) \end{aligned}$$

and

$$\begin{aligned} (R_0^{VZ})^2=K_{VZ}K_{ZV}=(\alpha pch_2^*k\psi ')/(r(\zeta +k\gamma )). \end{aligned}$$

Finally, there is a circuit of length 3, mites V infecting bees (which then enter Z) infecting other bees (which then enter Y) infecting mites (which then enter V), V$\rightarrow$Z$\rightarrow$Y$\rightarrow$V. We shall denote the partial basic reproduction number for this process as $R_0^{VZY}$, where

$$\begin{aligned} (R_0^{YVZ})^3=K_{YV}K_{VZ}K_{ZY}=(\alpha pch_2^*k\psi \tau ')/(r(\nu +k\gamma )(\zeta +k\gamma )). \end{aligned}$$

How many secondaries in class Y does a primary in class Y produce? We need to count the circuits in the graph in Fig. 6 that start at Y and end at Y. First, there is the circuit Y$\rightarrow$Y, with partial basic reproduction number $R_0^Y$. Then, there is the circuit Y$\rightarrow$V$\rightarrow$Y, but before returning from V to Y one may traverse the circuits V$\rightarrow$V and V$\rightarrow$Z$\rightarrow$V an indefinite number of times and in any order. The partial basic reproduction number for all these circuits is

$$\begin{aligned} (R_0^{YV})^2\left( 1 + (R_0^V + (R_0^{VZ})^2) + (R_0^V + (R_0^{VZ})^2)^2 + \cdots \right) . \end{aligned}$$

If $R_0^V+(R_0^{VZ})^2>1$, then this is unbounded and the disease invades whatever the other parameters of the system, while if $R_0^V+(R_0^{VZ})^2<1$ we may write this as $(R_0^{YV})^2/(1-R_0^V-(R_0^{VZ})^2)$. Finally, there is the circuit Y$\rightarrow$V$\rightarrow$Z$\rightarrow$Y, where from V one may again traverse the circuits V$\rightarrow$V and V$\rightarrow$Z$\rightarrow$V an indefinite number of times and in any order, to obtain invasion if $R_0^V+(R_0^{VZ})^2>1$ and $(R_0^{YVZ})^3/(1-R_0^V-(R_0^{VZ})^2)$ if $R_0^V+(R_0^{VZ})^2<1$. The condition for growth, that there are more secondaries than primaries, should be that either (i)

14 $$\begin{aligned} R_0^V + (R_0^{VZ})^2 > 1 \end{aligned}$$

or (ii) $R_0^V+(R_0^{VZ})^2<1$ and

15 $$\begin{aligned} R_0^Y + \frac{(R_0^{YV})^2 + (R_0^{YVZ})^3}{1 - R_0^V - (R_0^{VZ})^2} > 1. \end{aligned}$$

Note that this second inequality is always satisfied if $R_0^Y>1$. The expression on the left-hand side is not $R_0$, which is defined as the leading eigenvalue of a linear operator, but is the number of secondaries in class Y produced by a primary in class Y, and hence gives the same condition for growth that $R_0$ does. Indeed, the characteristic equation $P(\lambda )=0$ reduces to

$$\begin{aligned} P(\lambda ) =&-\lambda (\lambda - R_0^V)(\lambda - R_0^Y)\\&+ ((R_0^{YV})^2 + (R_0^{VZ})^2)\lambda + ((R_0^{YVZ})^3 - R_0^Y(R_0^{VZ})^2) = 0, \end{aligned}$$

and the condition $P(1)>0$, equivalent to $R_0>1$, reduces to

$$\begin{aligned} (R_0^Y - 1)(1 - R_0^V - (R_0^{VZ})^2) + (R_0^{YV})^2 + (R_0^{YVZ})^3 > 0, \end{aligned}$$

or (15) if $$R_0^V+(R_0^{YV})^2<1$$.

**Case (i)(a):** $$\rho = \sigma = 0$$, $$\tau = \tau ' = 0$$.

The parameters $\tau$ and $\tau '$ for horizontal transmission from bee to bee are small (so small that such transmission has not been included in most previous models). Let us assume that they are negligible, $\tau =\tau '=0$, while maintaining the assumption that there is no spontaneous transition, $\rho =\sigma =0$. The edges Y$\rightarrow$Y and Z$\rightarrow$Y in the graph disappear (so that $R_0^Y=(R_0^{YVZ})^3=0$), and it is easier to count the circuits from V back to V rather than from Y back to Y. They are V$\rightarrow$V, V$\rightarrow$Y$\rightarrow$V and V$\rightarrow$Z$\rightarrow$V. The condition that there are more secondaries than primaries, equivalent to the condition that $R_0>1$, is therefore

$$\begin{aligned} R_0^V + (R_0^{VZ})^2 + (R_0^{YV})^2 > 1. \end{aligned}$$

This is the inequality (15) with $R_0^Y=(R_0^{YVZ})^3=0$. All the terms are mite-related, and it is therefore the mites that drive overt disease in honey-bee colonies in this case, as we might have inferred from Sect. 3.1.1.

**Case(i)(b):** $$\rho = \sigma = 0$$, $$\zeta $$ large.

Here, we exploit the fact that the parameter $\zeta$ is large (since Z bees only survive 2 or 3 days compared to more than 20 days for X and Y bees). Then, the characteristic equation (13) has a root close to zero, $\lambda _1=\alpha pch_2^*(\psi \tau '-\psi '\tau )/(\varphi \psi (\zeta +k\gamma ))$. To leading order, the other two roots are the solutions of the quadratic

$$\begin{aligned} Q(\lambda ) = (\lambda - \theta )\left( \lambda - \frac{\tau }{\nu + k\gamma }\right) - \frac{k\varphi \psi }{r(\nu = k\gamma )}, \end{aligned}$$

which are $\lambda _2<\min \left\{ \theta ,\tau /(\nu +k\gamma )\right\}$ and $\lambda _3=R_0>\max \left\{ \theta ,\tau /(\nu +k\gamma )\right\}$. The characteristic equation has a single root greater than 1 if and only if $Q(1)<0$, or, in terms of partial basic reproduction numbers,

16 $$\begin{aligned} R_0^Y + \frac{(R_0^{YV})^2}{1 - R_0^V} > 1. \end{aligned}$$

#### Case (ii): $\rho$ and $\sigma$ Small, $\zeta$ Large

Now let us relax the assumption that $\rho =\sigma =0$, so spontaneous transition to overt disease does occur in bees and mites, as is realistic. To simplify the algebra, we shall retain the realistic assumption that $\zeta$ is large, so that we can neglect the Z class and the associated equation. Then, seeking steady states $(X^*,Y^*,U^*,V^*)$ of the X, Y, U and V equations in (6), we obtain $M=kN$ as before, and from the V equation

$$\begin{aligned} (r(1 - \theta ) + \sigma + \psi y^*)v^* = \sigma + \psi y^*, \end{aligned}$$

where $y^*=Y^*/N^*$, $v^*=V^*/M^*$, as usual. Then, from the Y equation, using $x^*+y^*=1$,

$$\begin{aligned} \rho (1 - y^*) - \nu y^* - k\gamma y^* + k\varphi (1 - y^*)v^* + \tau y^*(1 - y^*) = 0. \end{aligned}$$

Eliminating $v^*$ between these two equations, we obtain $C(y^*)=0$, where

$$\begin{aligned} C(y) =&\left( r(1 - \theta ) + (\sigma + \psi y) \right) \left( -\tau y(1 - y) - \rho (1 - y) + (\nu + k\gamma )y \right) \\&- k\varphi (1 - y)(\sigma + \psi y) = 0. \end{aligned}$$

This is a cubic with $C(0)=-(r(1-\theta )+\sigma )\rho -k\varphi \sigma <0$, $C(1)=(r(1-\theta )+\sigma +\psi ))(\nu +k\gamma )>0$, and there is a root $y^*$ of $C(y)=0$ in (0, 1). If $\rho$ and $\sigma$ are small, then so is *C*(0), so there is a root near zero, given by

$$\begin{aligned} y = \frac{r(1 - \theta )\rho + k\varphi \sigma }{r(1 - \theta )(-\tau + \nu + k\gamma ) - k\varphi \psi }, \end{aligned}$$

to leading order in $\rho$ and $\sigma$. If this expression is positive, it is the root in (0, 1), and is $O(\rho ,\sigma )$, so the steady state has low prevalence of overt infection Y (and hence V). If it is negative on the other hand it is not the root in (0, 1), the root in (0, 1) is not small, and there is an outbreak, high levels of overt infection Y and V. The condition for an outbreak is therefore

$$\begin{aligned} r(1 - \theta )(-\tau + \nu + k\gamma ) - k\varphi \psi < 0, \end{aligned}$$

or $(1-\theta )(1-\tau /(\nu +k\gamma ))-k\varphi \psi /(r(\nu +k\gamma ))<0$, or, in terms of partial basic reproduction numbers after dividing by $1-\theta$,

$$\begin{aligned} R_0^Y + \frac{(R_0^{YV})^2}{1 - R_0^V} > 1, \end{aligned}$$

exactly the condition for $R_0>1$ derived in (16). There is no standard basic reproduction number for this system, since there is no overt-disease-free steady state. However, the basic reproduction number $R_0$ for the system with $\rho =\sigma =0$ still has a role to play: there is an outbreak of overt infection if $R_0>1$. The condition for an outbreak without mites is the unrealistic condition $R_0^Y>1$, and overt disease is again mite driven.

## Conclusions

There are many differences in detail between our model and previously published ones. However there is one key difference driven by the biology that allows us new insight into the bee-mite-virus system. This is our distinction between covert and overt infection in bees and between low- and high-level infections in mites together with the associated possibility of spontaneous transitions between infected classes caused by replication of the virus within the bee or the mite population. These transitions have been widely reported in the literature, and the transition in mites in particular is recognised as crucial to the recent epidemiology of DWV. For DWV, it is also necessary to distinguish between those bees that gained their overt infection in brood cells and those that gained it in the hive, which are acutely and chronically overtly infected, respectively.

We have focussed our analysis on insights gained from exploring the dependence of $R_0$, the basic reproduction number, on model parameters and the origin of its constituent components. This presented particular challenges when the model system included spontaneous transition in bees or mites, but we addressed that by considering our model as a perturbation from a baseline system with no spontaneous transition, for which $R_0$ could be calculated.

We found it easier to determine the size of $R_0$ by analysing the weighted directed graph associated with *K*, the next-generation matrix. From this graph, we were able to extract expressions for the number of secondaries of class *i* produced by a primary of class *i*, and hence determine conditions for growth of the epidemic after a perturbation from the steady state, equivalent to $R_0>1$. These conditions are given in terms of the weights of the arcs in the directed graph, or equivalently in terms of partial basic reproduction numbers for circuits in the graph.

Further exploration of the conditions suggests that simple transmission of overt virus between adult bees could theoretically be sufficient to maintain overt infection in a colony (if $$R_0^Y>1$$), but this is unlikely with realistic parameters. Alternatively, it could be maintained solely by vertical transmission in mites coupled with their interactions with acutely overtly infected bees (if $$R_0^V+R_0^{VZ})^2>1$$), but this is also unlikely given that vertical transmission $$R_0^V$$ is a probability and therefore less than 1, and acutely infected adult bees die so quickly that their role in infecting mites is unlikely to be important. Therefore, it seems that circuits of transmission involving mites and chronically overtly infected bees ($$R_0^{YV})^2+(R_0^{YVZ})^3$$) must be involved.

If there are no mites, the condition for overt infection reduces to $R_0^Y>1$, which is unrealistic, in agreement with observations of DWV in colonies before the arrival of varroa.

In the perturbed system with spontaneous transitions (from covert to overt infection for bees and mites), if these transitions are rare, and making the realistic assumption for simplicity that acutely overtly infected bees are also rare, then the basic reproduction number $R_0$ for the unperturbed system still has a role to play. If $R_0<1$, then overt infection is rare, while if $R_0>1$ then there is an outbreak of overt infection. As in the unperturbed system, the condition for an outbreak without mites is the unrealistic condition $R_0^Y>1$, and overt disease is again mite driven.

Finally, if spontaneous transitions lead to high rates of overt infection in either bees or mites or both, then it is clear that overt disease will be prevalent independently of other processes. Spontaneous transitions in mites may be reasonably common. Spontaneous transitions in bees are in general rare, but neonicotinoids can lead to compromised immune systems and hence an outbreak of overt disease, even in the absence of mites.

Our work demonstrates the impact of distinct infection classes for both honey bees and their infection vector in maintaining viral infections within a honey-bee colony. It also highlights the importance of spontaneous transition between infection classes in both populations. As such, it provides an important theoretical contribution to inform future studies both theoretical and practical as we strive to find new approaches to preserve honey-bee populations worldwide.
